# Outcome of patients with septic shock and high-dose vasopressor therapy

**DOI:** 10.1186/s13613-017-0261-x

**Published:** 2017-04-20

**Authors:** Thomas Auchet, Marie-Alix Regnier, Nicolas Girerd, Bruno Levy

**Affiliations:** 1 Service de Réanimation Médicale Brabois, Pôle Cardiovasculaire et Réanimation Médicale, Hôpital Brabois, CHU Nancy, 54511 Vandoeuvre les Nancy, France; 2 Université de Lorraine, 54000 Nancy, France; 3 Anesthesiology and Surgical Intensive Care, Central Hospital, Nancy, University Hospital, Nancy, France; 4 INSERM, Centre d’Investigations Cliniques Plurithématique 1433, INSERM U1116, Université de Lorraine, CHRU de Nancy, F-CRIN INI-CRCT, Nancy, France; 5 INSERM, Groupe Choc, U1116, Faculté de Médecine, CHU Nancy, 54511 Vandoeuvre les Nancy, France

**Keywords:** Septic shock, Vasopressor, High-dose vasopressor, Refractory shock

## Abstract

**Background:**

Despite the dissemination of international guidelines, mortality from septic shock remains high. Norepinephrine is recommended as first-line vasopressor therapy with a target mean arterial pressure of 65 mmHg. High-dose vasopressor (HDV) may also be required. This study aimed to assess survival in patients with septic shock requiring HDV. We conducted a retrospective study of patients admitted between January 2008 and December 2013 to a 13-bed ICU for septic shock and receiving high-dose vasopressor therapy (defined by a dose >1 µg/kg/min). Primary outcome was 28-day mortality (D28). Secondary outcomes were 90-day mortality (D90), organ failure score (SOFA), duration of organ failure, duration and dosage of vasopressor agent and ischemic complications.

**Results:**

In our cohort of 106 patients, mortality reached 60.4% at D28 and 66.3% at D90. One in two patients died before D10. The weight-based mean dose of vasopressor (WMD) represented the best prognostic factor. Using a cutoff of 0.75 µg/kg/min, WMD was associated with mortality with a sensitivity of 73% and specificity of 74%. The mortality rate reached 86.4% when WMD was above the cutoff value and associated with a SOFA score >10. Digital or limb necrosis was documented in 6 patients (5.7%).

**Conclusions:**

In total, 40% of septic shock patients receiving high-dose vasopressor therapy survived at day 28 after admission. A WMD cutoff value of 0.75 µg/kg/min, associated with a >10 SOFA score, was a strong predictor of death. These results provide insights into outcome of refractory septic shock, showing that administration of high-dose vasopressor may indeed be useful in these patients.

## Background

Septic shock is the primary cause of admission and death in the intensive care unit [1]. Mortality exceeds 50% [1, 2] with most deaths caused by progressive hypotension despite hemodynamic support [3]. In the absence of myocardial dysfunction, hypotension is mainly caused by hypovolemia and hyporesponsiveness to vasopressors [4]. According to current guidelines [5], fluid replacement and vasopressor infusion, both guided by hemodynamic monitoring, must be titrated in order to increase mean arterial pressure to 65 mmHg and possibly to higher levels for patients with chronic hypertension [6, 7]. Norepinephrine is the recommended first-choice vasopressor with no recommendation for maximum dose and with early administration being associated with improved survival [8–11]. The pharmacodynamic effects of catecholamines are characterized by a linear increase in effect which is dependent on the logarithmic increase of the concentration [12]. Consequently, high doses of norepinephrine may be necessary to maintain mean arterial pressure (MAP) above 65 mmHg [13].

In the event of refractory septic shock, high-dose vasopressors may be used. Depending on the studies, high doses have been defined by a cutoff value ranging from 0.5 µg/kg/min to 2 µg/kg/min [14], although converging evidence has recently confirmed the dose of 1 µg/kg/min [15, 16]. However, mortality at these doses is substantial, from 80 to 100% [17–19]. Consequently, it is particularly difficult for practitioners to decide whether they should continue to increase norepinephrine. The ETHICUS study showed that the most common reason for ICU physicians to withhold or withdraw vasopressor administration was a lack of response to maximal therapy [20].

As a result, prognostic factors for patients with septic shock receiving HDV therapy are urgently needed.

In light of the above, the purpose of the present study was to (1) assess the outcome of patients receiving high-dose vasopressor therapy for septic shock and (2) determine the prognostic factors associated with mortality at these high doses.

## Patients and methods

### Patients

From January 2008 to December 2013, a retrospective, observational, non-interventional study was conducted in a 13-bed intensive care unit (ICU) at our university hospital (Hôpital de Brabois, Nancy, France). According to French legislation, neither informed consent nor approval by the ethics committee was needed for the use of routine data for an observational study.

All patients who were admitted for septic shock during the study period and had received high-dose vasopressor (HDV) therapy, i.e., epinephrine or norepinephrine, were studied. The norepinephrine-equivalent dose was based on prior studies: 1 µg epinephrine equivalent to 1 µg norepinephrine [15].

All patients admitted for septic shock were initially identified. From this group, those who received HDV as defined by a vasopressor dose ≥1 µg/kg/min for ≥1 h were ultimately selected. Patients were excluded on the following criteria: low vasopressor dose, diagnosis of septic shock 24 h prior to ICU admission or after the first day in ICU, septic shock complicated by cardiac arrest before admission.

Norepinephrine doses are expressed in terms of norepinephrine bitartrate.

### Septic shock management

Patients were treated according to standard procedures based on guidelines which were updated during the study period [11, 21]. The hemodynamic targets within the 6 first hours of intensive care were as follows: mean arterial pressure (MAP) ≥65 mmHg, urine output >0.5 ml/kg/h, SVc0_2_ > 70% and lactate clearance. All patients had a central venous catheter and were monitored using invasive blood pressure monitoring. Advanced monitoring devices included continuous hemodynamic analysis with the PiCCO system (continuous monitoring through pulse contour analysis with intermittent thermodilution measurement via the transpulmonary method) (Pulsion Medical System, Munich, Germany). Echocardiography was routinely performed in all patients.

Initial fluid therapy, mostly crystalloids, was guided by dynamic preload indices, with a minimal volume of 30 mL/kg. Norepinephrine was provided as first-line vasopressor. Epinephrine was only administered in one instance when it was introduced prior to admission in ICU. Epinephrine was replaced by norepinephrine as early as possible. Dopamine and vasopressin were not used. Inotropic treatment (dobutamine) was added in the presence of patent myocardial dysfunction: persistent signs of tissue hypoperfusion despite optimal fluid therapy and a MAP ≥ 65 mmHg, reduced arterial lactate clearance and SVc02 < 70%, echocardiographic and PiCCO system data. Stress-dose corticosteroids, namely 200 mg/24 h of hydrocortisone, were administered when adequate fluid resuscitation and vasopressor therapy were unable to restore hemodynamic stability.

A combination of empiric anti-infective therapy was administered within the first hour after recognition of septic shock, including antiviral and antifungal treatments when needed.

### Data collection

Demographic and clinical data were collected from medical charts and included general characteristics at baseline (age, gender, weight, prior medical history) as well as details of infection (community or hospital-acquired infection, site, pathogens). Organ failures were assessed by organ failure scores (sequential organ failure assessment (SOFA) and Simplified Acute Physiology Score 2 (SAPS2) at admission, SOFA at day 2 and day 5). Data pertaining to the treatment of organ failure (mechanical ventilation, renal replacement therapy) and its duration were also collected.

Hemodynamic data were extracted from medical charts. At ICU admission, the following variables were noted: heart rate, mean arterial blood pressure and cardiac index. The following variables regarding the initial treatment of septic shock were also collected: total fluid therapy, type of vasopressor therapy, use of inotropic treatment and stress-dose corticosteroids. Blood pressure and metabolic variables (pH, SVc02, serum lactate concentration, bicarbonate concentration) were collected at the start of vasopressor therapy and at every dose adjustment. At the end of vasopressor infusion, vasopressor dose variables (maximum dose, *t*_mean_ dose, cumulative dose) and duration of treatment were calculated.

Mortality was assessed during both ICU and hospital stay and thereafter at 28 and 90 days. Decisions relative to the withholding or withdrawing of life-sustaining therapies were documented.

The occurrence of ischemic events such as digital ischemia or mesenteric ischemia was noted.

#### Catecholamine variables

Several catecholamine variables (derived from epinephrine and norepinephrine data) were calculated:

- Maximum dose was the maximum infusion rate expressed in mg/h observed during ICU stay.
- Cumulated dose was the total amount of catecholamine received during ICU stay.
- Mean dose was calculated by dividing the cumulated dose by the cumulated duration of catecholamine administration.
- Mean initial 24-h dose was calculated by dividing the cumulated dose during the first 24 h by the cumulated duration of catecholamine administration during the first 24 h after ICU admission.
- Peak 6-h mean dose was obtained after dividing the hospital stay of each patient in 6-h intervals and identifying the 6-h interval (with at least 1-h administration of catecholamine during this interval) with the highest calculated 6-h mean dose.

### Statistical analysis

Quantitative baseline variables are expressed as mean ± SD or median (25, 75th quartile) and compared using one-sample *t* tests or Mann–Whitney tests as appropriate. Proportions are expressed as percentages and compared using Chi-square or Fisher’s exact tests as appropriate.

Survival probabilities were estimated with the Kaplan–Meier method.

Receiver operating characteristic (ROC) analyses were constructed to identify the prognostic value of each vasopressor dose variable as well as significant predictors of outcome in univariate analysis. Point estimates of sensitivity and specificity were reported for the best cutoffs identified within the ROC analyses.

Associations between vasopressor dose variables and 28-day mortality were assessed using logistic regression. The presence of an interaction between vasopressor dose variables and baseline SOFA was evaluated using an interaction term in three-variable logistic models (vasopressor variable, SOFA variable and vasopressor*SOFA variable). This search for interaction was prespecified given the interlinked nature of vasopressor dose and patient organ failure scores.

All statistical analyses were performed using SPSS for Windows (SPSS version 22, Chicago, Illinois). A *p* value <0.05 was considered to indicate statistical significance.

## Results

### Population characteristics

**Fig. 1:**
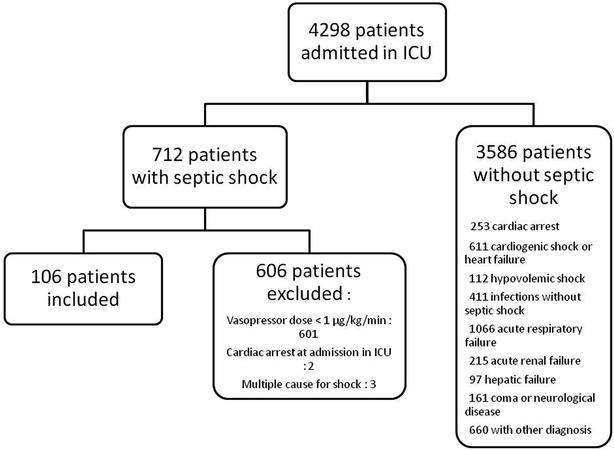
Flowchart of inclusion

A total of 106 patients were included during the study period (Fig. 1).

The main characteristics of the study population are summarized in Table 1. Slightly over half of the patients suffered from septic shock states due to pneumonia (51%). Other instances of septic shock were caused by endocarditis, urinary tract infections, abdominal infections, skin and soft tissue infections, catheter-related infections or gynecological infections. The source of infection remained unknown in 25% of patients.

**Table 1:** General characteristics of the study patients at baseline

Variables	All (*n* = 106)	Survivors at day 28 (*n* = 42)	Non-survivors at day 28 (*n* = 64)	*p*
Age (years), mean ± SD	62 ± 16	63 ± 14	59 ± 19	0.81
Sex [*n* (%)]				
Males	75 (70.8)	32 (76.2)	43 (67.2)	0.32
Comorbidities [*n* (%)]				
Immunosuppression	35 (33)	12 (28.6)	23 (35.9)	0.43
Cardiovascular disease	31 (29.2)	12 (28.6)	19 (29.7)	0.90
Respiratory disease	17 (16)	7 (16.7)	10 (15.6)	0.36
Renal disease	14 (13.2)	5 (11.9)	9 (14.1)	0.75
Obesity	15 (14.2)	8 (19.0)	7 (10.9)	0.24
Cirrhosis	12 (11.3)	1 (2.4)	11 (17.2)	0.03
Diabetes mellitus	30 (28.3)	13 (31.0)	17 (26.6)	0.62
Source of infection [*n* (%)]				
Community-acquired	55 (51.9)	25 (59.5)	30 (46.9)	0.20
Hospital-acquired	51 (41.1)	17 (40.5)	34 (53.1)	0.20

The mean length of ICU stay was 8 ± 14 days. For survivors, the length of ICU stay was 14 ± 22 days and length of hospital stay was 55 ± 74 days.

#### Septic shock treatment and vasopressor therapy

Most of the patients received norepinephrine as first-line vasopressor therapy (89%). Fourteen patients received norepinephrine and epinephrine (13%), and three patients received epinephrine only (2.8%). Twenty-eight patients received dobutamine as adjunctive therapy for cardiac failure (11 survivors and 17 non-survivors, *p* = 0.59). Details regarding organ failure and treatments are summarized in Table 2.

**Table 2:** Comparison of organ failures and septic shock treatment according to survival

Variables	All patients (*n* = 106)	Survivors at D28 (*n* = 42)	Non-survivors at D28 (*n* = 64)	*p*
Organ failure scores				
SOFA (admission) (mean ± SD)	12 ± 3	11 ± 3	13 ± 3	0.002
SOFA (day 2) (mean ± SD)	12 ± 4	10 ± 4	13 ± 4	0.01
SOFA (day 5) (mean ± SD)	8 ± 5	6 ± 5	12 ± 5	<0.001
Hemodynamic failure				
Mean arterial pressure (MAP) at admission (mmHg) (mean ± SD)	69 ± 20	69 ± 18	70 ± 21	0.69
Heart rate (beats per minute) (mean ± SD)	112 ± 26	106 ± 25	115 ± 27	0.12
Cardiac index (l/min/m^2^) (mean ± SD)	3.0 ± 0.86	3.0 ± 0.23	2.9 ± 0.13	0.64
Failure to achieve target MAP > 65 mmHg within 6 first hours [*n* (%)]	13 (12.2)	1 (2.4)	12 (18.8)	0.02
Initial fluid therapy (mL/kg) (mean ± SD)	29.7 ± 22.7	33.4 ± 23.6	28.2 ± 22.4	0.37
Stress-dose corticosteroids [*n* (%)]	89 (84)	40 (95.2)	49 (76.6)	0.04
Respiratory failure				
Mechanical ventilation [*n* (%)]	93 (88.6)	34 (81)	59 (93.7)	0.05
PaO2/FiO2 ratio (mmHg) [median (Q25–Q75)]	109 (77–200)	154 (87–228)	95 (68–167)	0.05
ARDS [*n* (%)]	43 (40.6)	11 (26.2)	32 (50)	0.01
Length of ventilation (days) [median (Q25–Q75)]	3.5 (1.3–8)	6.5 (3.8–10.3)	2 (1–7)	0.01
Renal failure				
Renal replacement therapy (RRT) [*n* (%)]	61 (57.5)	22 (52.4)	39 (60.9)	0.38
Metabolic parameters at admission				
pH (mean ± SD)	7.28 ± 0.15	7.29 ± 0.14	7.28 ± 0.16	0.62
Bicarbonates (mmol/l) (mean ± SD)	18.1 ± 5.6	18.0 ± 5.7	18.1 ± 5.6	0.96
Lactate (mmol/L) (mean ± SD)	4.8 ± 3.8	4.1 ± 2.6	5.4 ± 4.3	0.06
Lactate clearance H0–H6 (mmol/l) (mean ± SD)	0.15 ± 2.45	0.3 ± 2.0	0.0 ± 2.7	0.68
Maximal lactate concentration (mmol/l) (mean ± SD)	6.8 ± 4.4	5.3 ± 3.2	7.8 ± 4.9	0.004
SVO2 (%) (mean ± SD)	74.5 ± 11.4	75.4 ± 9.8	73.8 ± 12.6	0.569
Adequate antibiotherapy (mean ± SD)	98 (92.4)	39 (92.8)	59 (92.2)	0.85

In the study population as a whole, the weight-based mean dose of vasopressor (WMD) was 1.20 ± 1.05 µg/kg/min. Differences in doses and kinetics of norepinephrine administration between survivors and non-survivors are detailed in Table 3.

**Table 3:** Characteristics of vasopressor therapy according to survival

Characteristics of vasopressor therapy	All patients (*n* = 106)	Survivors at day 28 (*n* = 42)	Non-survivors at day 28 (*n* = 64)	*p*
Maximum dose (mg/h) (mean ± SD)	14.70 ± 11.3	10.55 ± 7.74	17.46 ± 12.51	0.002
Weight-based maximum dose (µg/kg/min) (mean ± SD)	3.28 ± 2.41	2.26 ± 1.55	3.97 ± 2.65	<0.001
Cumulative dose (mg) (mean ± SD)	291.33 ± 342.84	332 ± 438	263 ± 261	0.32
Weight-based cumulative dose (µg/kg) (mean ± SD)	3828 ± 4300	4222 ± 5389	3565 ± 3410	0.45
Mean dose (mg/h) (mean ± SD)	5.34 ± 4.73	3.32 ± 2.93	6.68 ± 5.22	<0.001
Weight-based mean dose (µg/kg/min) (mean ± SD)	1.20 ± 1.05	0.72 ± 0.61	1.52 ± 1.16	<0.001
First 24-h mean dose (mg/h) (mean ± SD)	7.01 ± 6.89	5.05 ± 3.90	8.30 ± 8.06	0.02
First 24-h weight-base mean dose (µg/kg/min) (mean ± SD)	1.57 ± 1.52	1.12 ± 0.87	1.86 ± 1.77	0.01
Peak 6-h mean dose (mg/h) (mean ± SD)	9.96 ± 6.89	7.70 ± 6.56	11.44 ± 6.75	0.006
Peak 6-h weight-based mean dose (mean ± SD)	2.26 ± 1.57	1.69 ± 1.33	2.64 ± 1.61	0.002
Duration of treatment (h) (mean ± SD)	84.66 ± 106.13	81.79 ± 48.30	86.57 ± 131.69	0.82

Mean vasopressor duration above 1 µg/kg/min was 84.7 ± 106.1 h, with no difference between survivors and non-survivors (81.8 ± 48.3 vs. 86.6 ± 131.7; *p* = 0.82).

#### Mortality during follow-up

**Fig. 2:**
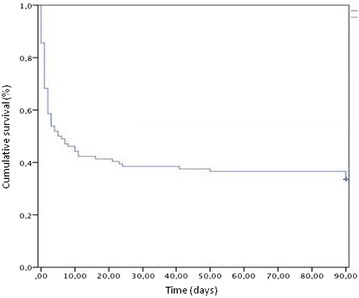
Survival analysis at 90 days: Kaplan–Meier curve

Among the 106 patients with septic shock requiring HDV, 42 survived at D28, 37 at D90. The mortality rate was 60.4% at 28 days and 65.1% at 90 days. Most deaths occurred within 10 days after admission in intensive care, as depicted in the Kaplan–Meier survival curve (Fig. 2). Withdrawal or withholding of care occurred in 32 of the 69 deaths (46%).

#### ROC analysis

**Fig. 3:**
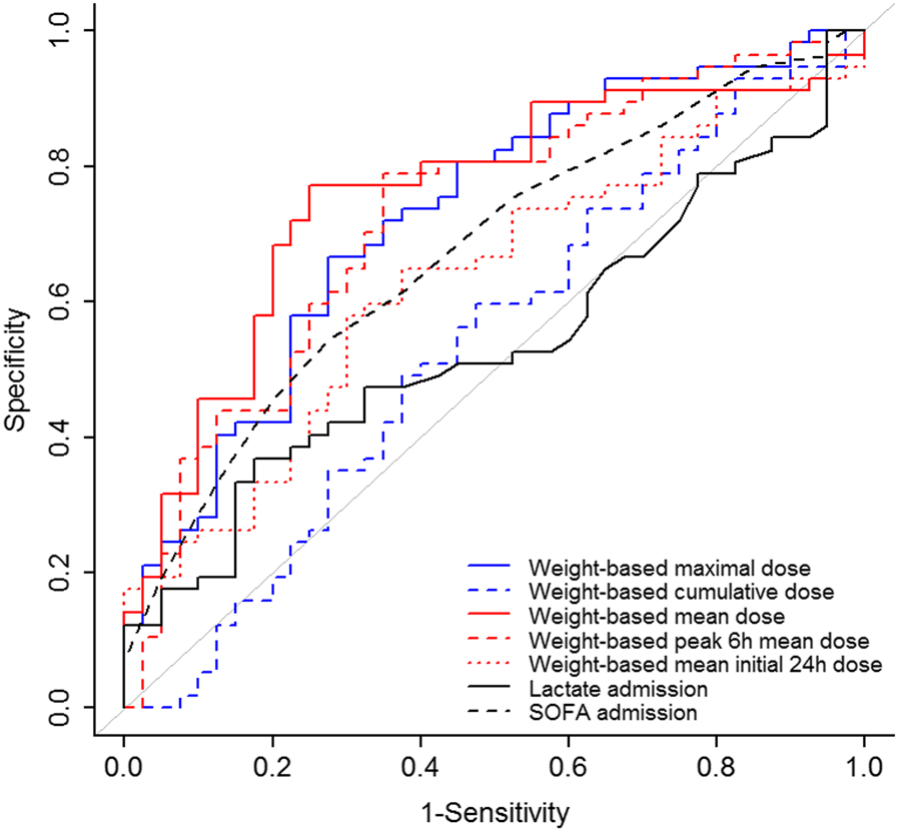
Receiver operating characteristic curves for vasopressor variables, SOFA, lactate and death

An ROC curve was used to determine the predictive ability of all vasopressor variables, lactate concentration, SOFA score and 28-day mortality (Fig. 3). The predictive ability of the WMD of norepinephrine [AUROC = 0.76 (0.66–0.86) *p* < 0.001] was greater than the other kinetic parameters of norepinephrine. The predictive ability of maximum weighted dose and the maximum 6-h mean dose was similar [respectively, 0.73 (0.63–0.83) *p* < 0.001 and AUROC = 0.73 (0.62–0.83), *p* < 0.001] although inferior to that of the WMD. As shown in Fig. 3, initial lactate level, cumulative norepinephrine dose, weight-based cumulative dose and initial 24-h mean dose were less predictive of 28-day mortality (all AUROC < 0.70).

Based on these ROC curves, the calculated cutoff value of 0.75 µg/kg/min for the WMD resulted in 73% sensitivity and 74% specificity for the likelihood of mortality. For the weight-based maximum dose (WMax), the ROC curves revealed a cutoff value of 2.30 µg/kg/min (with a sensitivity of 66% and specificity of 71%) and a cutoff value = 10 for the SOFA score (with a sensitivity of 64% and specificity of 74%). For the maximum weight-based 6-h mean dose, the ROC curves revealed a cutoff value of 1.38 µg/kg/min (with a sensitivity of 80% and specificity of 64%). The cutoff for the weight-based cumulative dose was 2300 µg/kg (with a sensitivity of 56% and specificity of 55%).

**Fig. 4:**
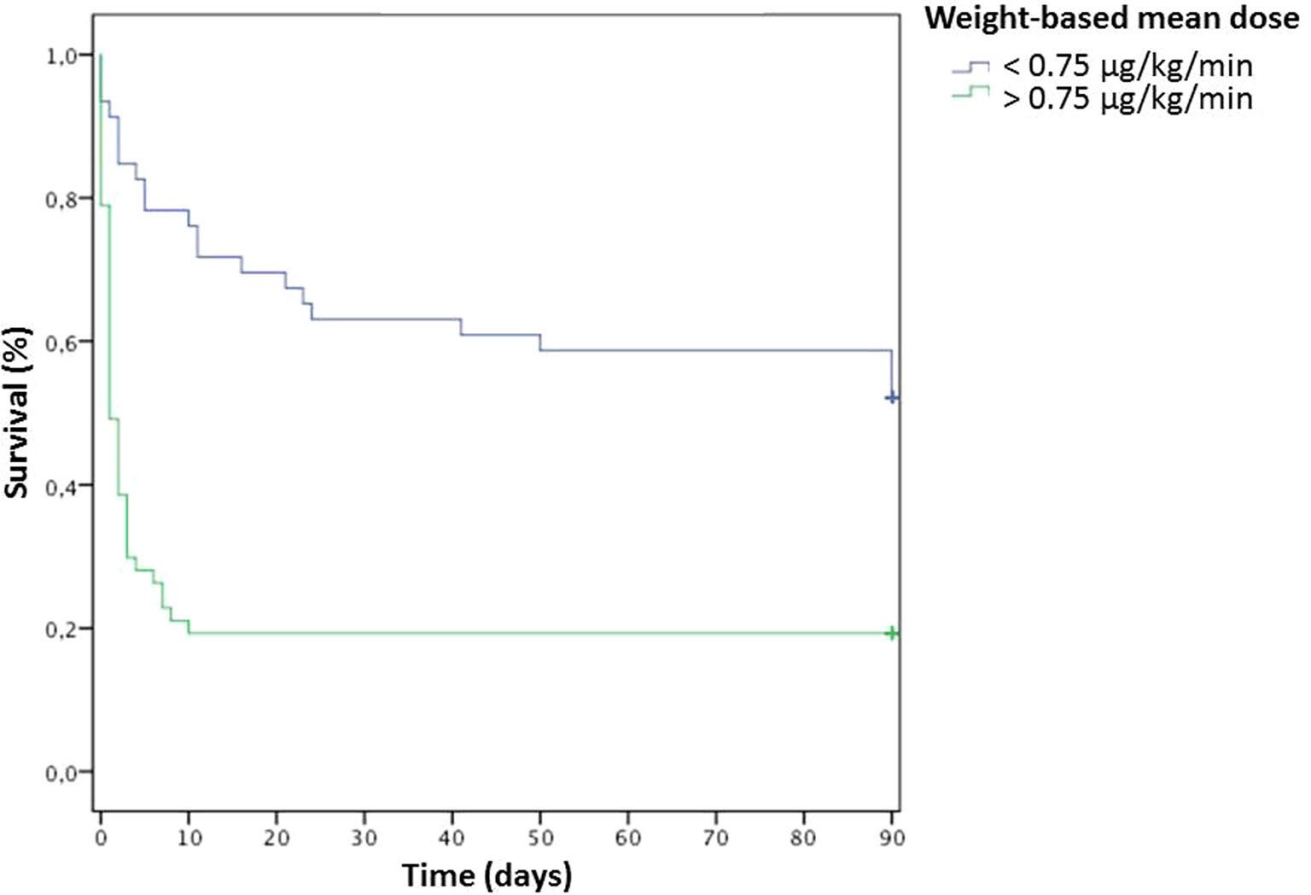
Survival analysis at 28 days according to a cutoff at 0.75 µg/kg/min of norepinephrine: Kaplan–Meier curve

At day 28, the mortality rate reached 80.4% for patients receiving a WMD ≥ 0.75 µg/kg/min and 35.4% for patients receiving a lower dose (*p* < 0.001) (Fig. 4).

#### Logistic regression analysis

After adjustment for SOFA score, logistic regression analysis revealed that the most significant variables for mortality in the ICU were WMD > 0.75 µg/kg/min [OR 6.04 (IC 2.42–15.06); *p* < 0.001], weight-based maximum dose >2.3 µg/kg/min [OR 3.95 (IC 1.64–9.54); *p* = 0.002] and maximum weight-based 6-h mean dose >1.38 [OR 5.74 (IC 2.33–14.13); *p* < 0.001]; weight-based cumulative dose (>2300 µg/kg) was not an independent predictor of mortality [OR 1.12 (IC 0.49–2.60); *p* = 0.79].

**Fig. 5:**
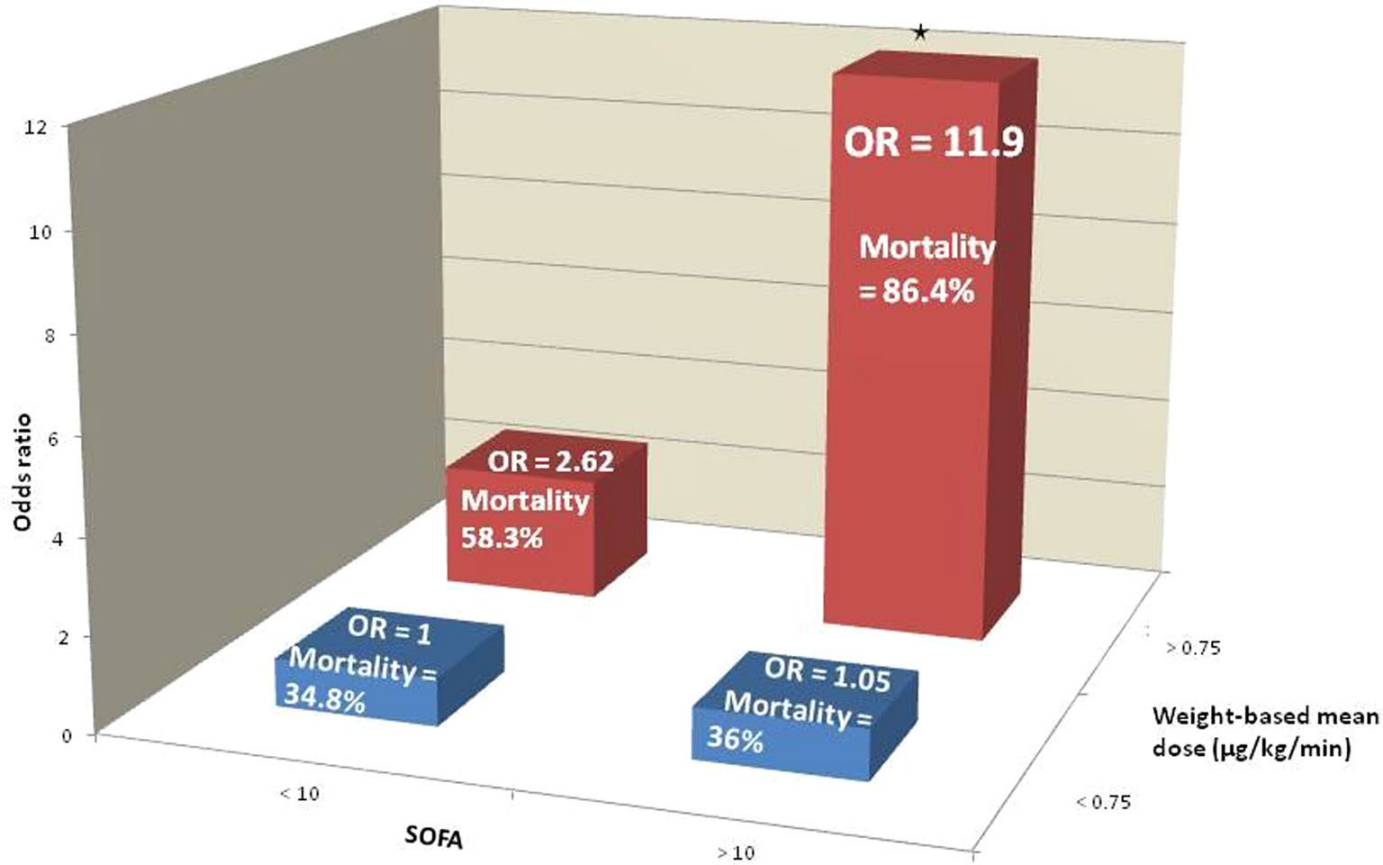
Correlation between WMD ≥ 0.75 µg/kg/min, SOFA > 10 and mortality

A significant interaction was identified between SOFA score >10 and WMD >0.75 µg/kg/min [OR 6.78 (IC 1.46–31.47), *p* = 0.015] resulting in a major increase in 28-day mortality risk in the presence of both parameters [OR 11.9 (IC 3.52–40.04); *p* < 0.001], with mortality reaching 86.4%. The interplay between WMD > 0.75 µg/kg/min and SOFA score >10 is shown in Fig. 5.

#### Sensitivity in patients without limitation of treatment

In the 71 patients without limitation of treatment, the WMD was similar to that observed for the entire group (1.23 ± 1.06 µg/kg/min). Likewise, weight-based maximum dose and weight-based cumulative dose were also similar to those observed for the entire group (respectively, 3.30 ± 2.49 and 353.30 ± 4458.19).

The predictive performance of WMD remained excellent [AUROC = 0.85 (0.75–0.95), *p* < 0.001].

In addition, after adjustment for SOFA score, logistic regression analysis in this population revealed that WMD > 0.75 µg/kg/min remained highly associated with outcome [OR 25.36 (IC 5.97–107.77); *p* < 0.001]. A similar pattern of risk was observed when dividing the population according to SOFA score (>10) and WMD (>0.75 µg/kg/min): 23/27 (85.2%) patients died when both parameters were present, whereas only 5/10 (50%) died when only the WMD factor was present and 2/17 (11.8%) when only the SOFA factor was present.

#### Ischemic complications

Digital or limb necrosis was documented in 6 patients (5.7%), 4 of whom necessitated surgical amputation and 1 died. Three patients suffered from bowel ischemia or infarction (2.8%) and subsequently died.

## Discussion

The main result of the present study is that in septic shock patients receiving high-dose vasopressor therapy, 28-day survival is elevated (40%) when compared to older published studies. The weight-based mean dose (WMD), associated with SOFA score at admission, represented the best prognostic factor.

### Study population

Only patients with septic shock requiring high-dose vasopressor (HDV) were included in the present study, as opposed to previous studies which included all types of shock [15, 17, 19, 22, 23]. In most instances, patients herein received norepinephrine exclusively, as recommended by the Surviving Sepsis Campaign [5]. Authors in previous studies have reported extremely high mortality rates in patients with shock and HDV, reaching upward of 94% [19]. Given the lack of common definition for refractory shock and high-dose vasopressor per se, there is considerable variability in administered dosages reported in the literature, ranging from 0.5 to 4 µg/kg/min [18, 19, 22, 24, 25]. Martin et al. [16] recently established a refractory dosage of 1 µg/kg/min with a 90% mortality rate at D90.

### Prognosis factors

The weight-based mean dose (WMD) of vasopressor was strongly associated with mortality with a cutoff of 0.75 µg/kg/min, to a greater extent than the weight-based maximum dose (WMax) with a cutoff of 2.30 µg/kg/min. The calculation of the WMD integrates each variation of vasopressor dose. Thus, WMD reflects the temporal evolution of hemodynamic status and is consequently a better prognostic factor than WMax. Accordingly, Kastrup et al. [22] found a significant difference between survivors and non-survivors for WMD although the authors did not assess the latter as a prognostic factor. Nonetheless, the performance of the various studied parameters (mean dose, maximum dose and cumulative dose) has been found to be systematically better when they are weight-based [26].

The maximum dose could indicate the severity of circulatory failure. In accordance with the findings of Kastrup et al. [22], it is our belief that the short-term administration of very high doses of catecholamines, especially during the first hours of septic shock, does not influence outcome and may be beneficial, particularly in instances of very low diastolic blood pressure which reflects a very severe hyporesponsiveness to vasopressors. This was nicely illustrated in a pharmacological study in which the authors found a linear relationship between epinephrine dose and response to treatment, without any saturation at high doses [27]. In our study, prolonged administration of high-dose vasopressor conversely indicated an uncontrolled circulatory failure regardless of etiology (vasoplegia, myocardial depression, hypovolemia) and was associated with poor outcome. Notwithstanding the above, WMax nevertheless remains a useful indicator and is directly available at bedside. The mortality rate reached 77.8% in our population when WMax was above 2.30 µg/kg/min, thereby confirming that a high WMax is associated with poor outcome [15, 17, 23]. Several authors identified a threshold of norepinephrine dose associated with 100% mortality: 2.22 µg/kg/min and 3.8 µg/kg/min in the Döpp-Zemel et al. [18] and Benbenishty et al. studies [17], respectively. However, some patients survived with doses greater than 4 µg/kg/min [24], thus underscoring that WMax should not be the sole factor in assessing patient prognosis.

### High-dose vasopressor and multiple organ failure

The kinetics of administration of vasopressor agents should not be interpreted individually. Organ failures, evaluated by the SOFA score [28], are strongly associated with mortality. In the present study, the predictive properties of vasopressor dose parameters (WMD, WMax) were superior to that of SOFA score alone. Moreover, there was a strong correlation between vasopressor dose and SOFA score. The combination of SOFA > 10 and WMD > 0.75 µg/kg/min was found to be a major risk factor associated with mortality (OR 11.9 ; IC 3.52-40.04; *p* < 0.001). This finding confirms the results of Abid et al. [29], who observed 100% mortality in patients with septic shock, high-dose vasopressor (dopamine and norepinephrine) and SOFA > 12. Brown et al. [15] and Döpp-Zemel et al. [18] both concluded that the mortality rate of patients in shock and HDV increased with higher organ failure scores (APACHE 2).

### Study limitations

Given its single-center retrospective nature, results obtained herein will need to be confirmed in a larger prospective multicenter cohort before any extrapolation can be made. Secondly, WMD was used in the present analysis. This variable, per se, can be calculated only when the patient dies or is weaned from vasopressor therapy. In order to utilize vasopressor dose as a prospective indicator of outcome, which could eventually guide clinical management, the use of refined bioinformatics techniques would be required. Indeed, in a given patient, numerous variables can be constructed from vasopressor doses at various time points, which are, by nature, highly correlated. In this setting, typical statistical approaches are usually inefficient, and bioinformatics or machine learning techniques are likely more appropriate. These new approaches could be used in future studies to enable information derived from vasopressor doses so as to guide the management of a given patient. Finally, the assessment of ischemic complications of HDV was a secondary objective of the study. Only a few patients suffered from ischemic complications and other confounding factors could have been analyzed such as pre-existing arteriopathy, impaired hemostasis and embolic diseases. As a result, no conclusions can be drawn regarding the ischemic consequence of high-dose vasopressor therapy.

## Conclusions

In the present study, 40% of septic shock patients receiving high-dose vasopressor therapy survived 28 days after admission. The weight-based mean dose, with a cutoff of 0.75 µg/kg/min, combined with SOFA score >10, was found to be a strong predictor of death. These results provide further insights regarding outcome in refractory septic shock.

## Abbreviations

SOFA: sequential organ failure assessment
SAPS 2: Simplified Acute Physiology Score 2
ICU: intensive care unit
HDV: high-dose vasopressor
WMD: weight-based mean dose of vasopressor
